# Peptide-Functionalized Dendrimer Nanocarriers for Targeted Microdystrophin Gene Delivery

**DOI:** 10.3390/pharmaceutics13122159

**Published:** 2021-12-15

**Authors:** Jessica Hersh, José Manuel Condor Capcha, Camila Iansen Irion, Guerline Lambert, Mauricio Noguera, Mohit Singh, Avinash Kaur, Emre Dikici, Joaquín J. Jiménez, Lina A. Shehadeh, Sylvia Daunert, Sapna K. Deo

**Affiliations:** 1 Department of Biochemistry and Molecular Biology, Leonard M. Miller School of Medicine, University of Miami, Miami, FL 33136, USA; jhersh@miami.edu (J.H.); mgn24@miami.edu (M.N.); mxs3086@miami.edu (M.S.); avinashkaur@outlook.com (A.K.); edikici@med.miami.edu (E.D.); j.jimenez@med.miami.edu (J.J.J.); sdaunert@med.miami.edu (S.D.); 2 The Dr. John T. McDonald Foundation Bionanotechnology Institute, University of Miami, Miami, FL 33136, USA; 3 Interdisciplinary Stem Cell Institute and Division of Cardiology, Department of Medicine, Leonard M. Miller School of Medicine, University of Miami, Miami, FL 33136, USA; jmcondor@med.miami.edu (J.M.C.C.); cii4@med.miami.edu (C.I.I.); glambert@med.miami.edu (G.L.); lshehadeh@med.miami.edu (L.A.S.); 4 Dr. Phillip Frost Department of Dermatology and Cutaneous Surgery, Leonard M. Miller School of Medicine, University of Miami, Miami, FL 33136, USA; 5 Clinical and Translational Science Institute, Leonard M. Miller School of Medicine, University of Miami, Miami, FL 33136, USA

**Keywords:** nanocarriers, gene delivery, targeted delivery, functional peptides, muscular dystrophy

## Abstract

Gene therapy is a good alternative for determined congenital disorders; however, there are numerous limitations for gene delivery in vivo including targeted cellular uptake, intracellular trafficking, and transport through the nuclear membrane. Here, a modified G5 polyamidoamine (G5 PAMAM) dendrimer–DNA complex was developed, which will allow cell-specific targeting to skeletal muscle cells and transport the DNA through the intracellular machinery and the nuclear membrane. The G5 PAMAM nanocarrier was modified with a skeletal muscle-targeting peptide (SMTP), a DLC8-binding peptide (DBP) for intracellular transport, and a nuclear localization signaling peptide (NLS) for nuclear uptake, and polyplexed with plasmid DNA containing the GFP-tagged microdystrophin (*µDys*) gene. The delivery of *µDys* has been considered as a therapeutic modality for patients suffering from a debilitating Duchenne muscular dystrophy (DMD) disorder. The nanocarrier–peptide–DNA polyplexes were prepared with different charge ratios and characterized for stability, size, surface charge, and cytotoxicity. Using the optimized nanocarrier polyplexes, the transfection efficiency in vitro was determined by demonstrating the expression of the GFP and the µDys protein using fluorescence and Western blotting studies, respectively. Protein expression in vivo was determined by injecting an optimal nanocarrier polyplex formulation to Duchenne model mice, mdx^4Cv^. Ultimately, these nanocarrier polyplexes will allow targeted delivery of the microdystrophin gene to skeletal muscle cells and result in improved muscle function in Duchenne muscular dystrophy patients.

## 1. Introduction

Many diseases are caused by single-gene mutations [1,2]. Treatment options for these diseases vary widely depending on disease presentation, and for some, the current treatment options fall short of a cure. Duchenne muscular dystrophy (DMD) is a devastating congenital disorder caused by an X-linked recessive mutation in the *DMD* gene, inducing an absence or decreased expression of dystrophin in skeletal muscle and cardiac muscle cells. The dystrophin protein links the cytoskeleton of muscle fibers to the extracellular matrix and serves as an important support protein in skeletal muscle cells [3]. The first signs of muscle weakness are detected in individuals, prominently males, when they are still very young. These patients, unfortunately, have a poor quality of life and an average life expectancy of 19 years [4].

While there is no cure for DMD, treatments exist which focus on trying to increase muscle strength through physical therapy and braces, steroids, and ventilatory support [4,5,6]. These treatments serve to delay the onset of muscle weakness. Gene therapies offer a promising alternative to treat these diseases, and although not many have been FDA-approved, they are being thoroughly studied in clinical trials [7,8,9]. Gene therapies offer promise for recessive gene disorders, some cancers, and some viral infections [9]. Broadly, gene therapies consist of introducing, removing, or modifying genetic material. The most common gene therapies studied for DMD use exon-skipping, which can only help patients with specific mutations. For example, eteplirsen (exon 51 skipping) is only applicable to 14% of patients [10]. In gene replacement therapy, a mutated or nonfunctioning gene is replaced with a functional one. DMD can be caused by any mutation of the dystrophin gene, making gene replacement therapy an ideal treatment choice.

It is important to note that in gene replacement therapy, a functional gene must be delivered to target cells. As such, to treat DMD with this technique, it is necessary to produce a functional dystrophin protein in skeletal muscle cells. However, there are major difficulties in delivering the dystrophin gene as it is the largest human gene at 2.2 mega-base pairs [11]. Therefore, many DMD gene therapy studies utilize the microdystrophin (*µDys*) gene, a shorter (3.7 kbp) functional version of the dystrophin gene, which has been demonstrated to cause DMD to present as a much less severe dystrophy with a longer life expectancy and higher quality of life [12,13,14]. While this therapy is promising, there are many barriers to effective gene delivery and transfection. The major steps include cellular uptake, cellular trafficking, and nuclear delivery. Overcoming these barriers results in high therapeutic efficacy.

Gene delivery requires a delivery vehicle which can bring the therapeutic gene to the therapeutic target. Viral vectors, adenovirus/adeno-associated in particular, have been studied for the longest time compared to other methods. DMD gene replacement therapies using adeno-associated virus (AAV) vectors are in preclinical and clinical studies [14,15,16,17,18]. However, viral vectors have limitations in that they can cause immunogenic effects and are not applicable to certain patient populations [19]. For example, three AAV-based phase I clinical trials are ongoing for delivery of the *µDys* gene [12], but all require subjects to have minimal to absent neutralizing antibodies, i.e., no prior exposure to any AAV. Because of this, nonviral vectors have been of growing interest. In particular, nanocarriers based on cationic vehicles, such as cationic polymers and liposomes, are explored for gene delivery due to their ability to electrostatically bind the negatively charged gene. One such example is the polyamidoamine (PAMAM) dendrimer. PAMAM dendrimers have advantageous properties such as uniform size distribution, easily modifiable surfaces, and the ability to bind and protect oligonucleotides from degradation [20,21,22,23]. These properties allow for the creation of smart nanocarriers that could be modified for targeted delivery of cargo such as DNA, RNA, proteins, and small molecules.

Despite the many beneficial properties of PAMAM dendrimers as nanocarriers, their progress in clinical applications has been limited due to challenges with cytotoxicity and biocompatibility [22,23]. However, recent studies have shown that these can be overcome with surface modification with organic molecules and peptides [24,25]. Peptide modification has the additional benefit of helping to overcome barriers to efficient transfection [20,21,26,27]. Methods such as phage display and identifying key functional sites from the known proteins have been deployed to provide researchers with an arsenal of functional peptides [28,29,30,31]. For gene delivery applications, peptides which target specific cells, facilitate cellular transport, and aid in nuclear uptake can surmount the major barriers to gene delivery [32].

Three targeting peptides were identified that can be incorporated into a PAMAM dendrimer nanocarrier loaded with the *µDys* gene in order to create a specialized therapeutic vehicle for targeted treatment of DMD. The three different peptides have three distinct targeting missions that enable the nanocarrier to reach the nucleus and deliver the *µDys* gene: (1) a skeletal muscle-targeting peptide (SMTP, ASSLNIA) that targets skeletal muscle cells and enhances cellular uptake [33,34]; (2) a dynein-binding peptide (DBP, CHHHKKKKETQTKKKHHHC) that facilitates intracellular transport by interacting with the DLC8 component of the dynein motor protein complex [35,36]; and (3) a nuclear localization sequence peptide (NLS, PKKKRKVEDPYC) that aids in nuclear uptake by binding to an adaptor protein in the nucleus [37,38].

In this work, a functionalized gene delivery nanocarrier polyplex that can overcome the barriers associated with transfection was developed and its ability to induce protein expression in vitro was demonstrated. An additional aim of this work was to show as a proof of principle that the dendrimer nanocarrier polyplex can induce protein expression in vivo in a relevant DMD animal model. If proven efficacious in delivery of genes and subsequent protein expression in skeletal muscles, this novel polyplex has the potential to treat Duchenne muscular dystrophy. By utilizing microdystrophin for gene replacement therapy, the polyplex can eliminate the limitations of traditional gene therapies for DMD that require identification of a specific mutation and can only treat a limited patient population. It may also overcome the drawbacks of AAV gene therapy, which include limited expression and liver toxicity [39,40]. Finally, in using a nonviral delivery vehicle, the multiple challenges associated with viral vectors can be avoided.

## 2. Materials and Methods

### 2.1. Dendrimer−DBP–NLS–Plasmid DNA Polyplex Formulation

#### 2.1.1. Main Polyplex Formulation Scheme

To form the nanocarrier polyplexes, 20% acetylated Generation 5 poly(amidoamine) (G5-PAMAM, 21st Century Biochemicals, Marlboro, MA, USA) dendrimers conjugated to an SMTP peptide (21st Century Biochemicals, ASSLNIA), DBP (Biomatik, Kitchener, Ontario, CA, CHHHKKKKETQTKKKHHHC) and NLS peptides (Biomatik, PKKKRKVEDPYC), and pDNA: pLv–has–µDys/EGFP—lentiviral plasmid containing the dystrophin gene (13,869 bp, pµDys) were used. This plasmid is commercially available and known to induce microdystrophin protein production in in vitro and in vivo studies. The pLv–has–µDys/EGFP was a gift from Jeffrey Chamberlain (Addgene plasmid #26810; http://n2t.net/addgene:26810, accessed on 17 August 2021; RRID: Addgene_26810) [41]. The components were combined at various charge ratios to form polyplexes. The charge ratios are defined as the ratio between the four components based on their surface charges and are written as G5 PAMAM–SMTP:DBP:NLS:pµDys. To determine the ratios of each component, first, the number of negative charges (phosphate groups) of pµDys was determine at a fixed concentration using Equation (1):

(1) $$\mathrm{Negative}\;\mathrm{charge}\;\mathrm{number}=\frac{\mathrm{Concentration}\times 6.022\times 10^{23}\times \mathrm{Number}\;\mathrm{of}\;\mathrm{negative}\;\mathrm{charges}\;\mathrm{per}\;\mathrm{plasmid}}{\mathrm{Molecular}\;\mathrm{weight}}$$

**Figure 1:**
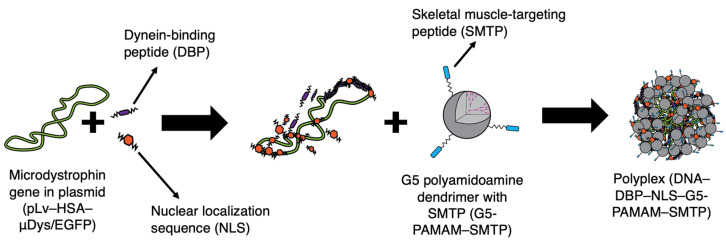
Components and synthesis order of peptide-modified dendrimer nanocarriers. Peptide structures are included in the Supplementary Information (Figure S1).

After determining the charge number, the same equation was used to determine the necessary concentration for different ratios using the number of positive charges per molecule for the G5-PAMAM–SMTP, DBP, and NLS molecules. For example, using equal volumes of 0.3 g/L G5-PAMAM–SMTP, 0.09 g/L DBP, 0.12 g/L NLS, and 0.15 g/L pµDys would result in a polyplex with a charge ratio of 5:1:1:1. All the solutions were prepared using 20 mM 4-(2-hydroxyethyl)-1-piperazineethanesulfonic acid (HEPES) buffer (pH 7.4). The polyplex synthesis is shown in Figure 1. The DBP and NLS peptides were incubated with pµDys for 45 min at room temperature after gentle vortexing. Following this, G5-PAMAM–SMTP was added, gently vortexed, and allowed to incubate for an additional 45 min.

#### 2.1.2. Polyplex Formulation with a Fusion Peptide

For some studies, a DBP–NLS fusion peptide, DBP–(GGS)2–NLS was used (Biomatik, CHHHKKKKETQTKKKHHHCGGSGGSPKKKRKVEDPYC). This fusion peptide was used in some experiments where the amount of the dendrimer varied in order to ensure a fixed ratio of DBP/NLS within the polyplexes.

#### 2.1.3. Polyplex Storage

In all the experiments, polyplexes were made fresh prior to analysis. The individual components were stored at −20 °C for long-term storage. Once the components were combined, the samples were stored at room temperature and were used within 2 h of their formation.

### 2.2. Gel Retention Assay

A 0.7% agarose gel was made using agarose powder (Lonza), 1× Tris–acetate–ethylenediaminetetraacetic acid (TAE) buffer (pH 8.3) and the GelRed dye (BIOTIUM). Samples were prepared at various charge ratios as described above, and the gel loading dye was added. Each sample was prepared with 1.5 µg of pµDys and each well was loaded with 0.5 µg of pµDys; 0.5 µg of naked pµDys was used as the negative control. The samples were loaded and run through the gel at 80 V for 1 h or until the loading dye had moved halfway down the gel and were imaged with UV light.

### 2.3. Protection from Serum Degradation Assay

Samples were prepared as described above, and naked pµDys was used as the positive control. Each sample was prepared using 4.5 µg pµDys. A negative control of 0.75 µg pµDys which was not exposed to serum proteases was also used. The samples and the positive control were incubated in complete cell medium (DMEM, Invitrogen, Waltham, MA, USA, supplemented with 10% fetal bovine serum) at 37 °C for 4 h. Following this, ethylenediaminetetraacetic acid (EDTA, Fisher Scientific, Waltham, MA USA,) was added to a final concentration of 5 mM and incubated for 5 min at room temperature to stop the reaction. Next, sodium dodecyl sulfate (SDS, VWR, Radnor, PA, USA) was added for a final concentration of 2.5% and incubated for 2 h at room temperature. Ethanol was added for overnight precipitation at −20 °C. The following day, the DNA was pelleted via centrifugation and resuspended in 20 µL water, from which 10 µL were loaded into the gel. The gel was prepared as described in Section 2.2, and the samples, the positive control, and the negative control were loaded and run through the gel at 120 V for 30 min or until the loading dye had moved halfway down the gel and were then imaged with UV light.

### 2.4. Size and Zeta Potential Characterization

Samples were prepared as described above using 4.5 µg pµDys, and their size and surface charge were analyzed using a Zetasizer NanoZS (Malvern Panalytical, Malvern, UK). The concentrated samples containing 4.5 µg pµDys were diluted to 1 mL using deionized water (Millipore) and analyzed in a cuvette (Sarstedt). Zeta potential measurements were performed using a dip cell (Malvern).

### 2.5. Cytotoxicity Assay

C2C12 cells (ATCC) were seeded in a 96-well plate at 2 × 10^3^ cells/well. Samples were prepared at various charge ratios as described above. The wells were treated with the various polyplexes 24 h after seeding, and no treatment was used as the control. Each treatment was repeated in triplicate. Multiple plates were used to obtain cytotoxicity results at 24 h, 48 h, and 72 h post-treatment. At each timepoint, an MTS assay (Promega, Madison, WI, USA) was performed according to the manufacturer’s protocol [42]. Briefly, the MTS dye was added to each well and allowed to incubate for 3 h in a cell incubator. After this time, absorbance measurements were taken at 490 nm using a CLARIOstar (BMG LABTECH, Ortenberg, Germany).

### 2.6. In Vitro Gene Delivery Studies

#### 2.6.1. Cell Lines and Transfections

C2C12 and HEK 293T cell lines were purchased from American Type Culture Collection (ATCC, Manassas, VA, USA). The cells were cultured in DMEM (Invitrogen), supplemented with 10% fetal bovine serum (Sigma, St. Louis, MO, USA), penicillin (100 units/mL), and streptomycin (100 μg/mL) in a 37 °C humidified incubator with 5% CO_2_. Samples were prepared as described above at various charge ratios using 4.5 µg pµDys. In the study described in Figure S2, 9 µg pµDys were used when doubling the treatment. Transient transfection of cells with mammalian expression vectors was performed using Lipofectamine 2000 (Invitrogen, Waltham, MA, USA) as the positive control according to the manufacturer’s instructions. Naked pµDys was used as the negative control. The cells were seeded in 12-well plates at 5 × 10^4^ cells/well. The media were replaced with Opti-MEM media (Gibco, Waltham, MA, USA) 24 h following seeding, and each well was treated with the samples containing 4.5 µg or 9 µg pµDys as described above.

#### 2.6.2. Live Cell Image Acquisition and Analysis

The plates were scanned with a 10× objective using an IncuCyte^®^ ZOOM live cell imaging system (Essen BioScience, Ann Arbor, MI, USA) 48 h post-transfection, after loading the plate into the system incubator, 25 images per well were obtained using the green and phase channels through the IncuCyte^®^ ZOOM software (2018A, Essen BioScience, Ann Arbor, MI, USA). The images were analyzed by dividing the total green object integrated intensity (green calibrated units (GCU) × μm^2^/image) values of each image by its corresponding total phase area (μm^2^/image) to obtain the normalized GFP expression (GCU) values per image. The GFP expression from all the images of each well and any replicate wells was averaged to obtain the group means, which represented the final GFP expression values and could be used for statistical analysis. Detailed protocols for the IncuCyte^®^ system and software operation have been described by our group [43,44].

### 2.7. In Vivo Gene Delivery

Animal procedures were approved by the University of Miami IACUC (approved April 15, 2021, protocol number 20-118-ad01). Wild-type, mdx^4Cv^ homo, and mdx^4Cv^ mice (JAX stock #002378, The Jackson Laboratory, Bar Harbor, ME, USA) [45,46] of 8–12 months of age were treated with the polyplex in a 50 µL volume via intramuscular injection (caudal muscle zone). The doses were based on the amount of pµDys and included 6.25 µg, 12.5 µg, 25 µg, and 50 µg pµDys. The animals were euthanized 48 h post-treatment. Caudal thigh, quadricep, tibialis anterior, and gastrocnemius muscles were snap-frozen in liquid nitrogen and saved in 4% paraformaldehyde. The muscles were homogenized in a RIPA lysis buffer supplemented with cOmplete™, Mini Protease Inhibitor Cocktail, under the manufacturer’s recommendations (Roche, Indianapolis, IN, USA).

### 2.8. Gene and Protein Expression

For gene expression (qPCR), RNA was extracted from cell lysates using a Mirvana Paris Kit (Thermo Fisher, Waltham, MA, USA), and we performed reverse transcription and cDNA amplification. Gene transcripts were quantified by means of qPCR using Taqman assays for human dystrophin (Taqman assay #Hs00758098_m1), EGFP (Taqman assay #Mr04097229_mr), GAPDH (Taqman assay #Mm99999915_g1), and a Fast qPCR master mix on an ABI 7900HT thermocycler. For each group, fluorescence was plotted against the number of cycles on a logarithmic scale, and the data were normalized to the endogenous GAPDH control. The normalized cycle thresholds (delta Cts) were converted to fold change relative to the control group. For protein expression (Western blotting), protein concentration from cell or tissue lysates from the pooled muscles was measured using a Bradford assay. Samples were prepared and separated using a 4–12% Novex mini 15-well gradient gel (Bolt System, Life Technologies, Waltham, MA, USA) and probed with the dystrophin (AB_2618171; DSHB, Iowa City, IA, USA), GFP (Ab290; Abcam, Cambridge, UK), and GAPDH (sc-25778, Santa Cruz Biotechnology, Santa Cruz, CA, USA) antibodies. Signals were detected by means of chemiluminescence (Femto, Thomas Scientific, Swedesboro, NJ, USA) on photographic films. Digitized images were analyzed using Image J (NIH, Bethesda, MD, USA). Protein band densitometry was normalized to that of GAPDH, and the averaged results were plotted as normalized densitometry units (n.d.u.).

### 2.9. Statistical Analysis

Data analysis and statistical analyses were performed using GraphPad Prism (GraphPad, La Jolla, CA, USA). All the experiments were performed in triplicate, and *p*-value < 0.05 was considered significant. Two-tailed Student’s *t*-test was used for two-group comparisons and one-way ANOVA was used for multiple comparisons. Post hoc analysis included Tukey’s tests for the cytotoxicity studies and Dunnett’s tests for the transfection studies.

## 3. Results and Discussion

### 3.1. Characterization of Nanocarrier Polyplexes

#### 3.1.1. Protection of pµDys

**Figure 2:**
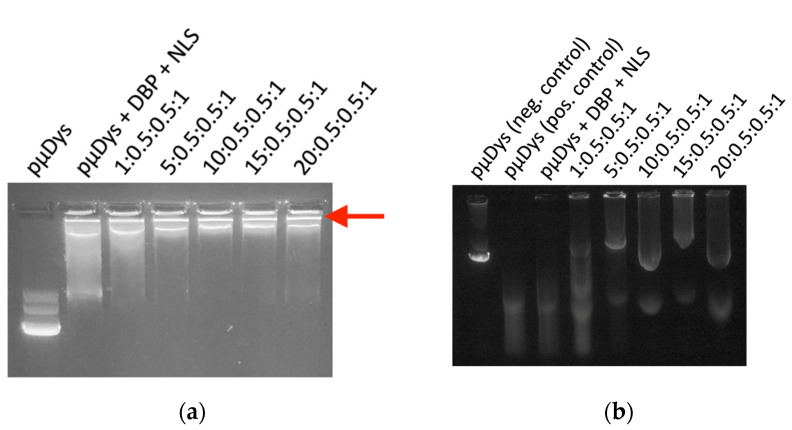
Gel images demonstrating the ability of G5-SMTP–DBP–NLS–pµDys to form polyplexes and encapsulate and protect pµDys. (**a**) Polyplexes of charge ratios 5:0.5:0.5:1 and higher are capable of fully binding pµDys, as indicated by the narrow band at the top of the gel (see arrow). (**b**) Polyplexes are capable of protecting pµDys from serum degradation, as shown by comparing the polyplex bands to the positive (lane 2) and negative (lane 1) control bands.

Polyplexes were prepared at different charge ratios, and their properties were evaluated to determine which of them have the most ideal characteristics. A detailed description of how charge ratios are determined and how to interpret the notation is in Section 2.1.1. The positively charged dendrimers ionically bind to the negatively charged pµDys, and thus it was necessary to determine the amount of the dendrimer needed to fully bind the gene. The polyplexes with a charge ratio of 5:0.5:0.5:1 (G5-PAMAM–SMTP/DBP/NLS/pµDys) and greater were able to fully bind pµDys. This is indicated by the fact that the polyplex, when run on an agarose gel, did not migrate much further from the loading well when compared to free unbound pµDys, which is able to migrate on the agarose gel. This shows that the plasmid encoding the *µDys* gene was successfully loaded onto the PAMAM dendrimer nanocarrier (Figure 2a). The migration of the naked pµDys indicates an unbound gene (Figure 2a, lane 1). With only the peptides complexed with pµDys or a polyplex formed with a low amount of the dendrimer (Figure 2a, lanes 2 and 3), there is complexation between pµDys and the peptides, although it does not bind as completely as with higher amounts of the dendrimer. It does, however, indicate that peptides are able to complex with pµDys. This is feasible since peptides are positively charged at pH below 10 and can bind negatively charged pµDys. At higher charge ratios, pµDys was strongly bound to the dendrimer, as indicated by the band in the wells (Figure 2a, lanes 4–7). Increasing the amount of the dendrimer in the polyplex enhanced the nanocarrier’s ability to hold cargo. This is attributed to the greater amount of positively charged molecules in the dendrimer which can more tightly bind a negatively charged pµDys–peptide complex.

Another important aspect of these polyplexes is their ability to protect pµDys from serum degradation. Certain molecules present in serum include proteases and specific endopeptidases that help degrade large circulating macromolecules. Upon the presence of serum proteases, the nanocarrier used requires a molecular composition that enables sufficient protection of the functional gene for efficient targeted delivery. As a result, the PAMAM dendrimer was used for its molecular composition and its attributes for electrostatic modifications. When exposed to serum proteases, pµDys in the polyplexes with charge ratios of 5:0.5:0.5:1 and higher was not degraded, as shown by the bands in the gel (Figure 2b). These bands match that of pµDys not exposed to any serum proteins (Figure 2b, lane 1). However, naked pµDys exposed to serum proteases was degraded (Figure 2b, lane 2). With only peptides (Figure 2b, lane 3) or with a low amount of the dendrimer (Figure 2b, lane 4), pµDys was not able to protect the gene from serum degradation. Together, these results demonstrate that polyplexes are capable of binding and protecting pµDys, making this nanocarrier suitable for therapeutic gene delivery.

#### 3.1.2. Size and Surface Charges of Polyplexes

**Figure 3:**
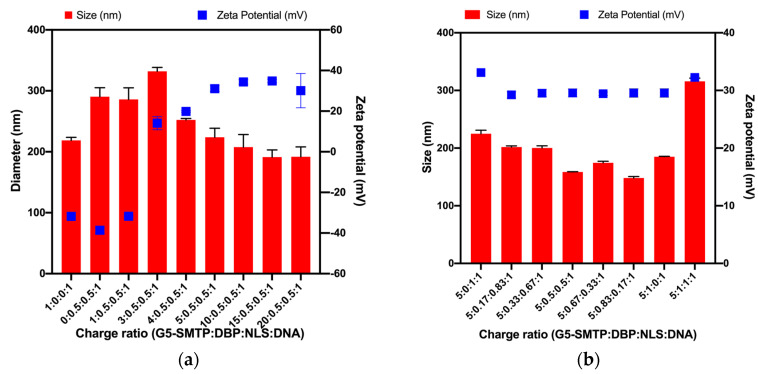
Dynamic light scattering and Zetasizer analysis of the polyplexes (G5-PAMAM–SMTP:DBP:NLS:pµDys). (**a**) Variations in size and surface charge with changes in the amount of the dendrimer. (**b**) Variations in size and surface charge with changes in the ratio of the DBP and NLS peptides while keeping the dendrimer/pµDys ratio constant at 5:1.

Although dendrimer nanocarriers have diameters of ~5.4 nm [47,48], the polyplexes which combine all of the components are larger. In order to be used for in vivo gene delivery applications, the polyplex diameter must be less than 1000 nm, and ideally less than 200 nm for the maximum delivery efficiency [49]. The surface charge must be positive and sufficiently high to prevent aggregation and instability, yet sufficiently low to prevent toxicity [50,51,52,53,54]. The effect of the amount of the dendrimer on size and surface charge was explored by varying the dendrimer/pµDys ratios from 0:1 to 20:1 and using a DBP–NLS fusion peptide as described in Section 2.1.2. The polyplexes were of an appropriate size, measured using DLS, and surface charge based on zeta potential, with the sizes decreasing and surface charges increasing as the charge ratio increased (Figure 3a). While all the polyplexes were below 1000 nm, only those of the 5:0.5:0.5:1 ratio and greater were around 200 nm or smaller. Similarly, charge ratios of 5:0.5:0.5:1 and greater had the most ideal surface charges for preventing aggregation. Positive surface charge also aids in cell attachment and internalization [55].

To further explore the effect of the various components on size and surface charge, a dendrimer/pµDys ratio of 5:1 was maintained and the amount of DBP and NLS in the polyplexes was varied. The surface charge was uniform and stable across all the ratios and sufficiently high to prevent aggregation (Figure 3b). The variations in the amount of the peptide have little influence on the surface charge. The size, however, varied without any discernable trend, with certain ratios polyplexes forming at more ideal sizes than with others (Figure 3b). In particular, 5:0.5:0.5:1 and 5:0.83:0.17:1 resulted in the smallest sizes. From this, it can be concluded that polyplexes have suitable characteristics required for cellular delivery.

### 3.2. In Vitro Performance of Nanocarriers

#### 3.2.1. Cytotoxicity in C2C12 Mouse Skeletal Muscle Cells

**Figure 4:**
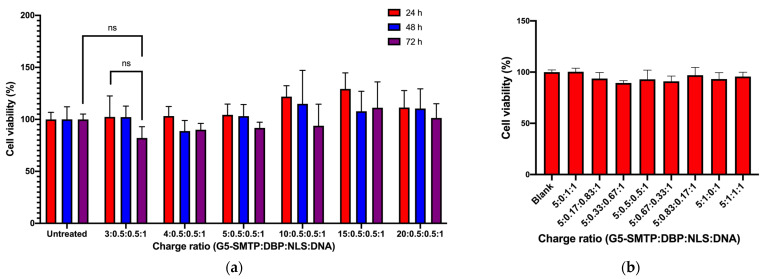
Cytotoxicity analysis of the polyplexes from the MTS proliferation assay using C2C12 cells. (**a**) Variations in cytotoxicity with different amounts of the dendrimer. Cell viability was monitored over 3 days. (**b**) Variations in cytotoxicity due to changes in the ratio of the DBP and NLS peptides while keeping the amount of the dendrimer the same.

The toxicity of the polyplexes was determined using an MTS proliferation assay for various charge ratios over three days in the cells of interest, skeletal muscle cells. In the first study, the amount of the dendrimer was varied, and a DBP–NLS fusion peptide was used as described in Section 2.1.2. Overall, cytotoxicity is very low, and the charge ratio has minimal effect on cytotoxicity. There was no significant difference between any of the treatments compared to the control or the other experimental groups. The polyplexes have slightly increasing toxicity over time, but there is no significant decrease in cell viability (Figure 4a). Additionally, the effect of altering the ratio of peptides within the polyplexes was looked at in a short-term study, and it was found that there was no significant difference in cytotoxicity across all of the charge ratios (Figure 4b). The lowest cell viability of all the treatments was found in the polyplex formed at a ratio of 5:0.33:0.66:1. This was not significantly lower than the blank control, and was a high value of 89%, which indicates minimal toxicity. Overall, these polyplexes have demonstrated minimal cytotoxicity, making them suitable for further in vitro studies to determine the transfection efficiency as well as for in vivo studies to determine therapeutic efficacy.

#### 3.2.2. Transfection in HEK 293T and C2C12 Cells

Numerous studies were conducted to investigate the ability of the dendrimer nanocarrier polyplex to transfect pµDys into cells. For this study, the HEK 293T cell line was used initially to compare differences in transfection efficiency caused only by the varying charge ratios. HEK 293T cells were chosen as they are commonly used for transfection and gene delivery studies. In this work, different charge ratios were studied, and the EGFP expression also encoded in pµDys was monitored. After 48 h, the treatments were further analyzed by means of Western blotting to detect the microdystrophin protein. Lipofectamine 2000 was used as the positive control. Lipofectamine is a cytotoxic delivery vehicle that is used as a standard for exogenous gene delivery only in in vitro and ex vivo transfection studies, but not in vivo [56,57]. However, it serves as a good positive control for in vitro studies. Differences in transfection from altering the amount of the dendrimer in the complexes was investigated using charge ratios ranging from 1:0.5:0.5:1 to 20:0.5:0.5:1. Similarly, the effect of changing the ratio of peptides was determined by maintaining the ratio of G5-PAMAM–SMTP/pµDys at 5:1. This charge ratio was chosen because of its transfection abilities, as well as its ideal size, surface charge, and biocompatibility.

**Figure 5:**
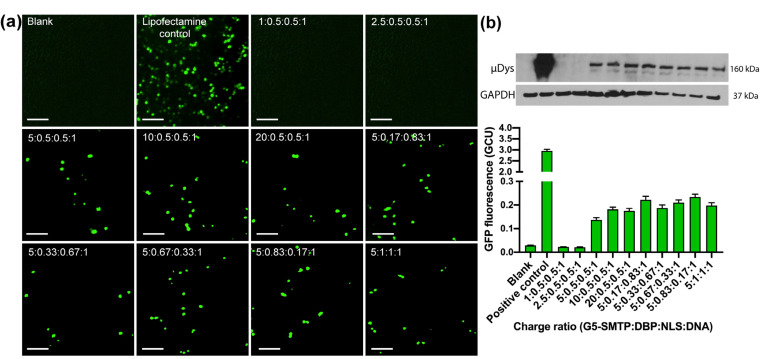
HEK 293T cells were treated with the polyplexes formed at various charge ratios, and the EGFP and µDys expression was quantified after 48 h. (**a**) Representative fluorescent images for EGFP expression. Scale bar: 300 µm. (**b**) Quantification of EGFP fluorescence and Western blotting for microdystrophin. EGFP signal was normalized to the cellular area. The blots follow the same loading and charge ratios as the graph.

Throughout the studies that investigated charge ratios, it was determined through EGFP fluorescence and Western blotting that only charge ratios of 5:0.5:0.5:1 and greater were able to induce protein expression in cells, as indicated by EGFP expression and Western blot analysis (Figure 5b). Low charge ratios of 1:0.5:0.5:1 and 2.5:0.5:0.5:1 did not produce a microdystrophin band (Figure 5b). Fluorescent images confirmed that low charge ratios do not express EGFP (Figure 5a). The low charge ratios were associated with negative zeta potentials, making them anionic (Figure 3a). The anionic polyplexes were less effective in transfection than their cationic counterparts. This could in part be due to the anionic polyplexes being less effective at protecting the gene cargo from serum degradation (Figure 2b). Additionally, it has been demonstrated that cationic molecules can achieve better cellular uptake and lysosomal escape compared to anionic molecules [58,59,60]. Other nanoparticle transfection studies have produced similar results in which cationic polyplexes transfected more efficiently than neutral or negatively charged polyplexes [61,62].

When varying the peptide ratio, the results demonstrated that all the variations of the peptide ratio produced EGFP and microdystrophin expression, although there was no trend. The fluorescent images demonstrate how the varying peptide ratios affect EGFP production (Figure 5a), while the associated fluorescence quantification allows for accurate comparisons between the treatments (Figure 5b). Repetitions of this experiment showed that addition of the peptides enhanced the transfection, although there was still no trend between the peptide ratio and the transfection efficiency (Figure S2). Western blotting analysis supported the conclusions drawn from the EGFP data.

Throughout all the transfection studies in the HEK 293T cells, the treatments performed an order of magnitude below the positive control, so two methods for increasing the transfection levels of the treatments were identified: further optimization of the nanocarrier polyplexes and increasing the treatment dose. In doubling the volume of a treatment, there was a significant increase in EGFP expression (Figure S2). This suggests that the in vivo efficiency could be increased by finding the optimal dose as well as the optimal charge ratio.

**Figure 6:**
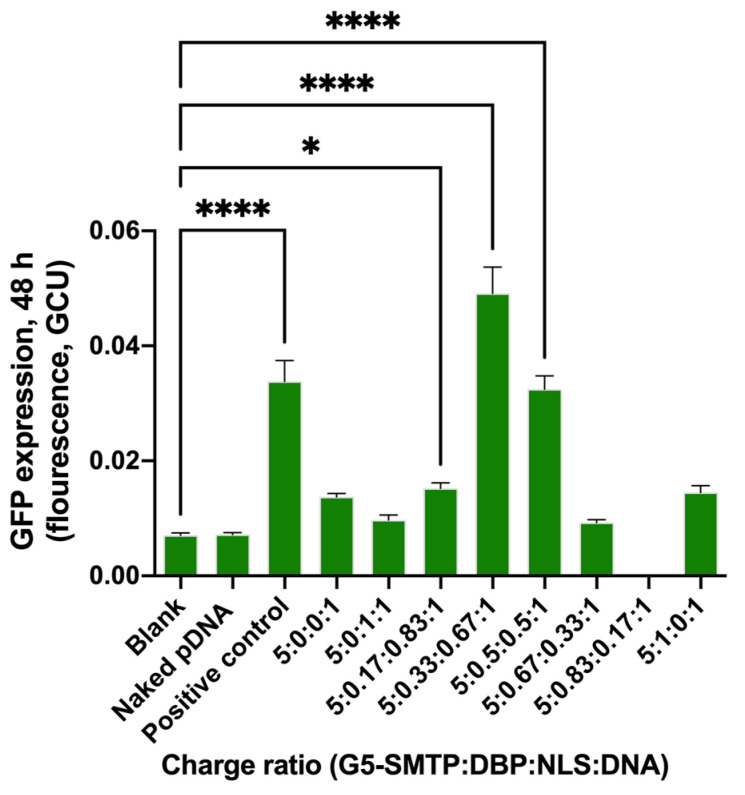
Nanocarrier polyplexes can deliver µDys–GFP in C2C12 cells. C2C12 cells were treated with the polyplexes formed at various charge ratios and the EGFP expression was quantified by measuring fluorescence after 48 h. Lipofectamine 2000 was used as the positive control. * denotes significance *p* ≤ 0.05, **** denotes significance *p* ≤ 0.0001.

The treatment efficiency in the cells of interest, C2C12 mouse muscle myoblasts, was explored. As mentioned above, most of the in vitro transfection studies were performed in HEK 293T cells because myoblast cells have inherent limitations with transfection [63,64,65,66]. However, this study was performed to demonstrate feasibility of the approach in the target cells. Charge ratios which had been tested in the HEK 293T cells were used to treat the C2C12 myoblasts to check the treatment efficiency in the cells of interest after 48 h. Many treatments performed significantly better than the negative control, and charge ratios of 5:0.33:0.67:1 and 5:0.5:0.5:1 performed at similar or higher levels to the positive control of Lipofectamine (Figure 6). Overall, the EGFP expression was lower in the C2C12 cells than in the HEK 293T cells due to known issues with transfecting myoblast cultures [63,64,65,66]. Despite this, EGFP expression was at least as good as or better than the positive control in the cells of interest, suggesting that active targeting and functional peptides enhance cellular uptake. Similarly, the known issues in in vitro myoblast transfection will not be an issue in vivo as skeletal muscles are the most abundant tissue and thus an ideal target for this gene therapy. These results supported moving forward to test the treatment in vivo based on the successful delivery to the nucleus and expression of both EGFP and µDys.

### 3.3. In Vivo Performance of Nanocarriers

The positive outcome of the in vitro data led to a small pilot animal study to determine in vivo gene delivery. The aim of that study was to determine whether the expression of µDys in vivo in skeletal muscle cells can be obtained using a targeted nanocarrier. This study was performed using a DMD mouse model, female mdx^4Cv^ mice (*n* = 5). This model is one of the most commonly used in DMD studies, and the phenotype is caused by a chemically induced point mutation [46]. Mdx^4Cv^ mice have 10-fold fewer revertants than other DMD mouse models, making them more ideal for gene therapy studies [67].

**Figure 7:**
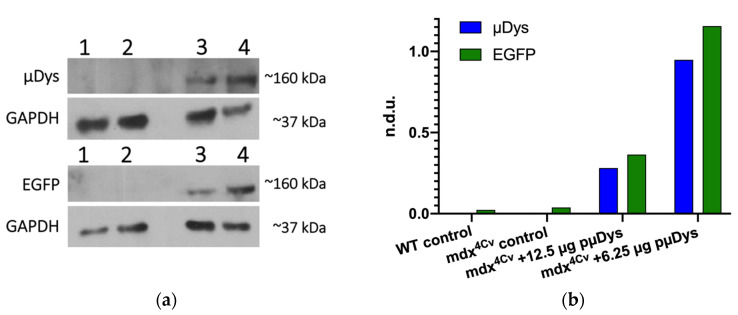
Microdystrophin delivery in vivo. A 1-year-old female mouse with DMD (mdx4Cv) was treated in both legs with 50 μL of concentrated polyplex (12.5 µg pµDys in the left leg, 6.25 µg pµDys in the right leg). After 48 h, protein analysis was performed on leg tissues. (**a**) Western blot analysis of the pooled leg muscles revealed μDys and EGFP expression. (**b**) The µDys and EGFP bands were normalized to the GAPDH bands and the ratios were quantified. 1. Untreated wild-type. 2. Untreated mdx4cv. 3. Treated mdx4cv—left leg muscles. 4. Treated mdx4cv—right leg muscles. Dystrophin (MANEX1011B; DSHB, AB-2618171), EGFP (Abcam, Ab290); n.d.u: normalized densitometry units.

Since the charge ratio 5:0.5:0.5:1 was found to be one of the optimal ratios, different doses of the treatment were tested. The charge ratio of 5:0.5:0.5:1 was selected over other effective charge ratios to have both peptides present in equal amounts. The doses were based on the amount of pµDys and included 6.25 µg, 12.5 µg, 25 µg, and 50 µg of pµDys. All the other polyplex components were adjusted to achieve a 5:0.5:0.5:1 ratio. Figure 7 shows a representative study where a mouse was treated with 12.5 µg pµDys in the left leg (Figure 7, lane 3) and 6.25 µg pµDys in the right leg (Figure 7, lane 4) via intramuscular injection. Western blot analysis for µDys and EGFP demonstrated targeted delivery and in vivo efficacy when compared to both the untreated wild-type control (Figure 7, lane 1) and the untreated Mdx^4Cv^ control (Figure 7, lane 2). The wild-type control would have dystrophin, but not microdystrophin. The Mdx^4Cv^ control served to demonstrate the lack of microdystrophin, dystrophin, and EGFP in the DMD mouse model, while the wild-type control confirmed that there are no nonspecific bands at 160 kDa, the MW of microdystrophin.

Across all the studies, the lower doses of 6.25 and 12.5 µg pµDys proved to be delivered at a higher efficiency than the larger doses of 25 and 50 µg pµDys; qPCR analysis in one study demonstrated greater gene expression for the 12.5 µg dose compared to the 25 µg dose (Figure S3). However, this could be due to aggregation which occurred in the formation of the higher doses. Because the injection volume was fixed at 50 µL, the larger doses had a higher sample concentration, resulting in increased particle collision events and thus aggregation. A potential solution to this challenge could involve administering more than one dose, namely opt for administration of repeated lower doses in order to achieve a greater total pµDys amount.

As already described, most microdystrophin studies in progress involve the use of viral vectors, and these studies have progressed to preclinical and clinical studies. Preclinical studies have involved mice as well as larger mammals such as dogs and demonstrated similar functional efficacy [14,15,16,17]. Results from a clinical trial published in 2020 found no T cell responses and strong expression of microdystrophin proteins and showed functional improvement based on North Star Ambulatory Assessment scores [18]. These studies have demonstrated efficacy of microdystrophin as a therapeutic for DMD. However, the viral vectors used in these studies pose limitations in addition to immunogenic challenges [19]. In addition, AAV carriers require a promoter, and these are not always tissue-specific [68,69,70]. In contrast, a nanocarrier-based polyplex does not require a promoter and can target skeletal muscle cells selectively.

Other nonviral methods can also overcome the disadvantages of AAVs, and there are nonviral gene delivery studies in progress for DMD. However, these studies utilize morpholinos and exon skipping as opposed to gene replacement therapy with microdystrophin [71]. While these methods are effective for specific mutations, they are limited to certain patient populations. Nonviral microdystrophin methods have used nonviral gene vectors [72,73], antibodies [74], and transplanting stem cells [75], but some of these studies have not advanced much in recent years. In that regard, the methodology described in this paper provides a nonviral delivery method for microdystrophin with promising in vitro and in vivo results.

## 4. Conclusions

In this study, a gene delivery nanocarrier which targets the cells of interest, enhances cellular uptake, facilitates cellular trafficking, and aids in nuclear transport of the gene of interest was designed and prepared. The core of this nanocarrier is a G5-PAMAM dendrimer which was polyplexed with a plasmid containing the gene for µDys as a therapeutic cargo and incorporates the SMTP, DBP, and NLS peptides for targeting. The resulting polyplex was demonstrated to have ideal characteristics with regard to optimal size, surface charge, pµDys-binding capabilities, and minimal cytotoxicity. It was determined that a charge ratio of 5:0.5:0.5:1 had the minimum amount of a dendrimer nanocarrier required to achieve the target size, surface charge, and gene cargo protection. Altering the ratio of the DBP and NLS peptides in the polyplex similarly produced polyplexes of the target size and surface charge. None of the charge ratios tested caused significant toxicity in the cells of interest.

The goal of this work, to demonstrate that the polyplex has the capability to induce protein translation in vitro and in vivo, was achieved. Similarly to polyplex characterization, a minimum charge ratio of 5:0.5:0.5:1 was necessary for transfecting the HEK 293T cells. The polyplexes were also capable of transfecting at the level of the positive control in skeletal muscle cells, the cells of interest. Finally, preliminary in vivo data demonstrated that the polyplexes induced protein production in a DMD mouse model. With this successful proof of concept, further studies involving extensive analysis of the biodistribution, histology, immunological effects, and functional efficacy will need to be performed in a larger-scale animal study.

As described, this nanocarrier polyplex was able to induce microdystrophin protein expression, and thus has a potential to serve as a therapeutic agent for Duchenne muscular dystrophy by delivering the µDys gene to replace the mutated gene in patients with the disease. Additional therapeutic applications of the dendrimer nanocarrier can be achieved by customization with different targeting peptides and therapeutic cargoes to create treatments for other diseases that need therapeutic gene delivery.

## Data Availability

Data is contained within the article or supplementary materials.

## Supplementary Materials

The following are available online at https://www.mdpi.com/article/10.3390/pharmaceutics13122159/s1, Figure S1: Peptide structures, Figure S2: Transfection efficiency of the increased dose, Figure S3: Quantitative PCR gene expression in different leg tissues.
