# Supplementary material for: Peptide-Functionalized Dendrimer Nanocarriers for Targeted Microdystrophin Gene Delivery
Source: Pharmaceutics. 2021 Dec 15;13(12):2159. doi: 10.3390/pharmaceutics13122159 (PMC8708248; doi:10.3390/pharmaceutics13122159)
Supplement: Supplementary file 1 [file pharmaceutics-13-02159-s001.zip › pharmaceutics-1468323-supplementary.pdf]

pharmaceutics

MDPI

Supplementary Materials: Peptide-Functionalized Dendrimer Nanocarriers for Targeted Microdystrophin Gene Delivery

Jessica Hersh, José Manuel Condor Capcha, Camila Iansen Irion, Guerline Lambert, Mauricio Noguera, Mohit Singh, Avinash Kaur, Emre Dikici, Joaquín J. Jiménez, Lina A. Shehadeh, Sylvia Daunert and Sapna K. Deo

1. Peptide Structures

Peptide Structures. The peptides used in this study include the skeletal muscle targeting peptide (SMTP, ASSLNIA), the dynein binding peptide (DBP, CHHHKKKKETQTKKKHHHC), and the nuclear localization sequence peptide (NLS, PKKKRKVEDPYC). Their structures are depicted in Figure S1.

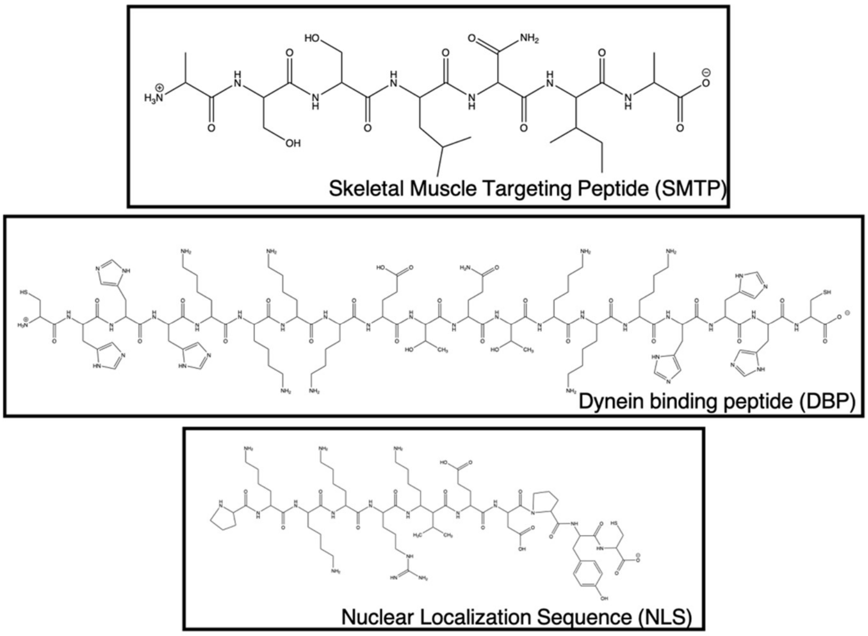

Figure S1. Peptide structures of the SMTP, DBP, and NLS peptides.

2. Additional Transfection Studies

Transfection Efficiency of Increased Dose. eGFP expression was quantified and normalized to a positive control as part of an *in vitro* study replicate (Figure S2). Representative images are included which demonstrate that addition of peptides enhances eGFP expression. In addition to replicating treatments, this experiment tested doubling the treatment volume for one charge ratio. The results showed significantly higher eGFP expression in the increased dose when compared to the regular dose, indicating that greater doses can result in increased transfection.

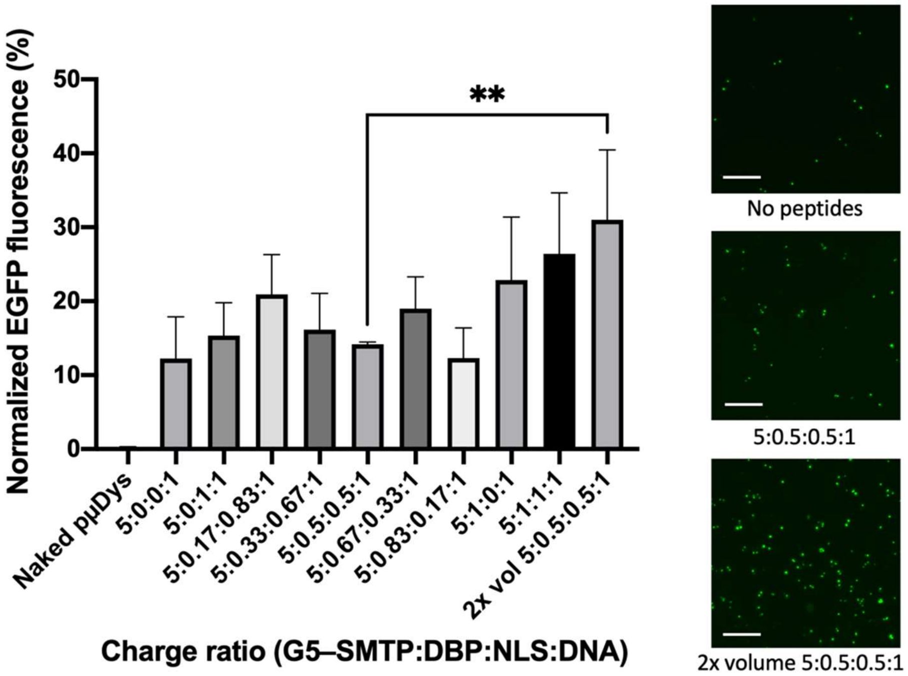

Figure S2. Transfection Efficiency of Increased Doses. eGFP expression for various charge ratios in HEK 293T cells using charge ratios which had been repeatedly tested, as well as an increased dose of one treatment. Scale bar: 150 µm. ** denotes significance $p \leq 0.01$.

3. qPCR Analysis of Leg Tissues

Gene Expression in Different Tissues. We performed qPCR and determined the fold change for both µDys and EGFP gene expression in different leg tissues. For the wild type and mdx4Cv controls, only the quadriceps muscle was analyzed. The results show that for both treatments, the gene expression was largely localized to the injection site, the caudal muscle. Interestingly, there was increased expression in the gastrocnemius muscle in the leg which received the lower dose, indicating the treatment was able to spread other leg tissues. Other leg muscles did not show much gene expression for either treatment. Although the EGFP gene had greater fold changes compared to the µDys gene, the relative fold change between the different treatments and different muscles was consistent. While the lower dose did have a greater fold change than the higher dose, this phenomenon could be explained by aggregation as described in the main text.

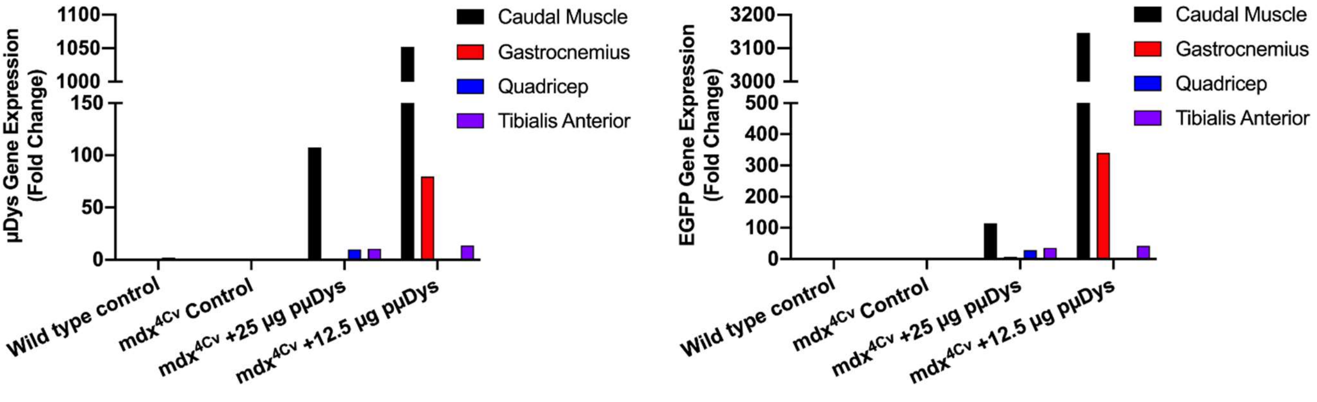

(**a**) (**b**)

Figure S3. Gene expression in various leg muscles determined by qPCR. For both treatments, expression was largely localized to the caudal muscle. Both genes measured showed similar pattern of expression. (**a**) Expression of µDys gene. (**b**) Expression of EGFP gene.
